# Novel *LAMA2* variants identified in a patient with white matter abnormalities

**DOI:** 10.1038/s41439-020-0103-5

**Published:** 2020-05-26

**Authors:** Keiko Yamamoto-Shimojima, Hiroaki Ono, Taichi Imaizumi, Toshiyuki Yamamoto

**Affiliations:** 1 Japan Society for the Promotion of Science (RPD), Tokyo, Japan; 2 Institute of Medical Genetics, Tokyo Women’s Medical University, Tokyo, Japan; 3 Tokyo Women’s Medical University Institute for Integrated Medical Sciences, Tokyo, Japan; 4 Department of Pediatrics, Hiroshima Prefectural Hospital, Hiroshima, Japan; 5 Department of Pediatrics, St. Marianna University School of Medicine, Kawasaki, Japan

**Keywords:** Genetics research, Medical genetics

## Abstract

Comprehensive genomic analysis was performed in a patient with mild psychomotor developmental delay, elevated creatine kinase, and white matter abnormalities. The results revealed biallelic pathogenic variants in the gene related to merosin-deficient congenital muscular dystrophy, NM_000426.3(LAMA2):c.1338_1339del [p.Gly447Phefs*7] and c.2749 + 2dup, which consist of compound heterozygous involvement with predicted loss-of-function and splicing abnormalities.

The laminin-alpha2 gene (*LAMA2*; MIM#156225) located on 6q22.33 is responsible for congenital muscular dystrophies, merosin-deficient congenital muscular dystrophy type 1A (MDC1A; MIM#607855) and late-onset limb-girdle muscular dystrophy-23 (MIM#618138), in association with an autosomal recessive trait. *LAMA2* consists of 65 exons and encodes 3122 amino acids producing a 343.9-kDa protein^1^.

*LAMA2* pathogenic variants were first identified in patients with MDC1A^2^, which is characterized by difficulty walking, hypotonia, proximal weakness, hyporeflexia, and white matter intensities on brain magnetic resonance imaging (MRI). Most of the pathogenic *LAMA2* variants identified in patients with severe, neonatal-onset MDC1A have been associated with loss-of-function effects. In comparison, missense variants are associated with milder CMD with partial *LAMA2* deficiency^3^. This indicates the existence of a genotype-phenotype correlation in *LAMA2*-related disorders. Furthermore, atypical *LAMA2*-related disorders have also been reported previously^4^.

Recently, we encountered a patient with mild psychomotor developmental delay and brain white matter abnormality. Novel *LAMA2* variants were identified in this patient.

He is a 3-year-old boy born at the 38^th^ week of gestation, with a birth weight of 2610g and both parents healthy and unrelated. There was no family history of neuromuscular disorders. At 12 months of age, the child was referred to the hospital due to motor developmental delay; he could not crawl or stand by himself. Owing to muscular hypotonia, blood examination was performed, and elevated creatine kinase (CK; 873 IU/µL) was detected. Echocardiogram showed no abnormality in his heart. Screening for inborn errors of metabolism showed no abnormality. At 24 months, he started to walk alone. At that time, he could speak only a few meaningful words, indicating mild psychomotor developmental delay. At 30 months of age, brain MRI was performed, and T2-high signal was diffusely observed in the white matter (Fig. 1a-c). At present, his growth parameters are within normal limits. However, speech and social development delays were observed. He can use a fork but cannot use chopsticks. He cannot hold a button. He does not understand abstract concepts and the meaning of numbers. According to the Enjoji developmental test, his developmental quotient was evaluated as 58^5^. All subordinate items were low on average. CK levels remained elevated. For precise diagnosis of this patient, a comprehensive genetic analysis was performed.

**Fig. 1:**
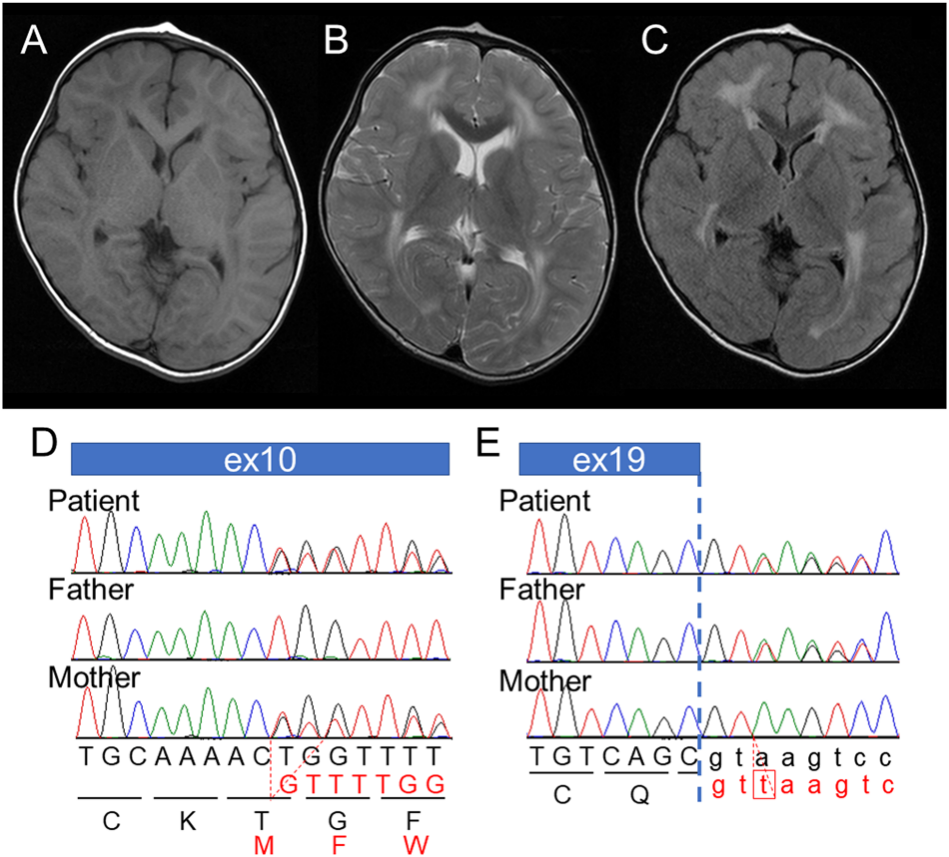
Patient information. **a**–**c** Brain MRI findings. T1 (**a**) and T2 (**b**) weighted axial images and a T2-flair axial image (**c**). T2- and T2-flair images show high signal intensity in the white matter (**b**, **c**). **d**, **e** Electropherograms of Sanger sequencing results. A 2-bp deletion is shown in the patient and his mother (**d**). 1 bp (t) is duplicated in the splicing donor site in the patient and his father (**e**).

This study was performed in accordance with the Declaration of Helsinki. We obtained permission from the ethics committee of the institution. The family of this patient provided written informed consent after going through careful genetic counseling with regard to the handling of genetic information and possible incidental findings. Blood samples were obtained from the patient and his parents. DNA was extracted using a QIAamp DNA extraction kit (QIAGEN, Hilden, Germany). Next-generation sequencing (NGS) was performed to screen single-nucleotide variants using the TruSight One v1.0 sequencing panel (Illumina, San Diego, CA, USA), as described previously^6^. The extracted data were mapped to a reference genome (GRCh37/hg19) using BWA Enrichment v1.0 cloud software (Illumina) and then annotated and filtered by using Variant Studio software (Illumina). Standard genomic PCR, reverse-transcription PCR (RT-PCR), and Sanger sequencing were performed in accordance with previous studies^7^.

After filtering the data, pathogenic variants were identified in *LAMA2*; NM_000426.3:c.1338_1339del [NP_000417.2:p.Gly447Phefs*7] and NM_000426.3:c.2749 + 2dup. In the data, there was no other possible candidate variant related to leukodystrophies and muscular disorders. These variants were confirmed by Sanger sequencing. Parental samples were also analyzed to detect the origin of the variants. The results showed that c.1338_1339del and c.2749 + 2dup were inherited from his mother and father, respectively. The 2-bp deletion was considered pathogenic since it is predicted to cause premature termination. In comparison, a 1-bp duplication in the splice donor site, c.2749 + 2dup, was identified in the SNP database as rs759144210. The frequency of this variant in the ExAC database was extremely low at 1/121332 (0.000008), and the ClinVar database suggested this to be of uncertain significance.

Because this splice donor site duplication (c.2749 + 2dup) may cause splicing abnormalities, we tried to analyze this possibility by using mRNA expressed in EB-transformed leukocytes. However, *LAMA2* RNA was not detected, which could be due to the extremely low expression level in this sample. Instead, we used in silico software, including Human Splicing Finder (http://www.umd.be/HSF3/HSF.shtml), Alternative Splice Site Predictor (ASSP; http://wangcomputing.com/assp/index.html), Fruit Fly Splice Predictor (http://www.fruitfly.org/seq_tools/splice.html), and NetGene2 Server (http://www.cbs.dtu.dk/services/NetGene2/). All results predicted loss of the original donor site (Supplemental Figs. S1–4). Alternatively, new donor sites may be used, and exon 19 may be extended in such an occurrence. These extended exons are predicted to cause a frameshift. When this was evaluated through the ACMG recommendation, this variant fulfilled only PM3, PP3, and PP4, indicating “uncertain significance”. However, we consider the genetic relevance of this variant (c.2749 + 2dup) to the specific clinical features of this patient.

Although there is only a report on *LAMA2*-related disorders from Japan^8^, the common variant has never been reported. Furthermore, the two variants identified in this study are not included in the Leiden Muscular Dystrophy Pages (http://www.dmd.nl/LAMA2). Thus, c.1338_1339del and c.2749 + 2dup were considered rare but related to the clinical findings in this patient.

In this patient, motor developmental delay was mild, and muscular weakness was not obvious. Thus, leukodystrophy was first suspected as a possible candidate diagnosis rather than muscular disorders. Finally, a molecular diagnosis of *LAMA2*-related disorder was made through comprehensive genomic analysis. Generally, loss of function in *LAMA2*-related disorders is related to severe manifestations of muscular weakness. On the other hand, mild motor developmental delay is related to missense variants. In this patient, c.1338_1339del is related to loss-of-function. Thus, one more variant (c.2749 + 2dup), predicted to cause splicing abnormality, may not be related to complete loss-of-function.

The patient also showed mild delay in language and social development. Although most patients with *LAMA2*-related disorders have normal intelligence, some children have been reported to show moderate intellectual disability^9^. Therefore, retrospective analysis of clinical information suggested no contradiction with the molecular diagnosis of *LAMA2*-related disorder in this patient.

Although the findings of the brain MRI in patients with *LAMA2*-related disorder resemble those of leukodystrophy, there is no evidence of dysmyelination in this disorder. Because *LAMA2* is expressed in the brain blood vessels, it is suggested that *LAMA2* may be important for the selective filtration capability of the blood-brain barrier and that the dysfunction of *LAMA2* may cause impaired selective filtration, leading to the leakage of plasma components and damage to the CNS^9,10^.

## Supplementary information

Supplemental Figure S1-4

## Data Availability

The relevant data from this Data Report are hosted at the Human Genome Variation Database at 10.6084/m9.figshare.hgv.2847, 10.6084/m9.figshare.hgv.2850.
