# Supplementary material for: Novel LAMA2 variants identified in a patient with white matter abnormalities
Source: Hum Genome Var. 2020 May 26;7:16. doi: 10.1038/s41439-020-0103-5 (PMC7248065; doi:10.1038/s41439-020-0103-5)
Supplement: Supplementary file 1 — Supplemental Figure S1-4 [file 41439_2020_103_MOESM1_ESM.pdf]

Supplemental Figure S1. In-silico analysis using Human Splicing Finder

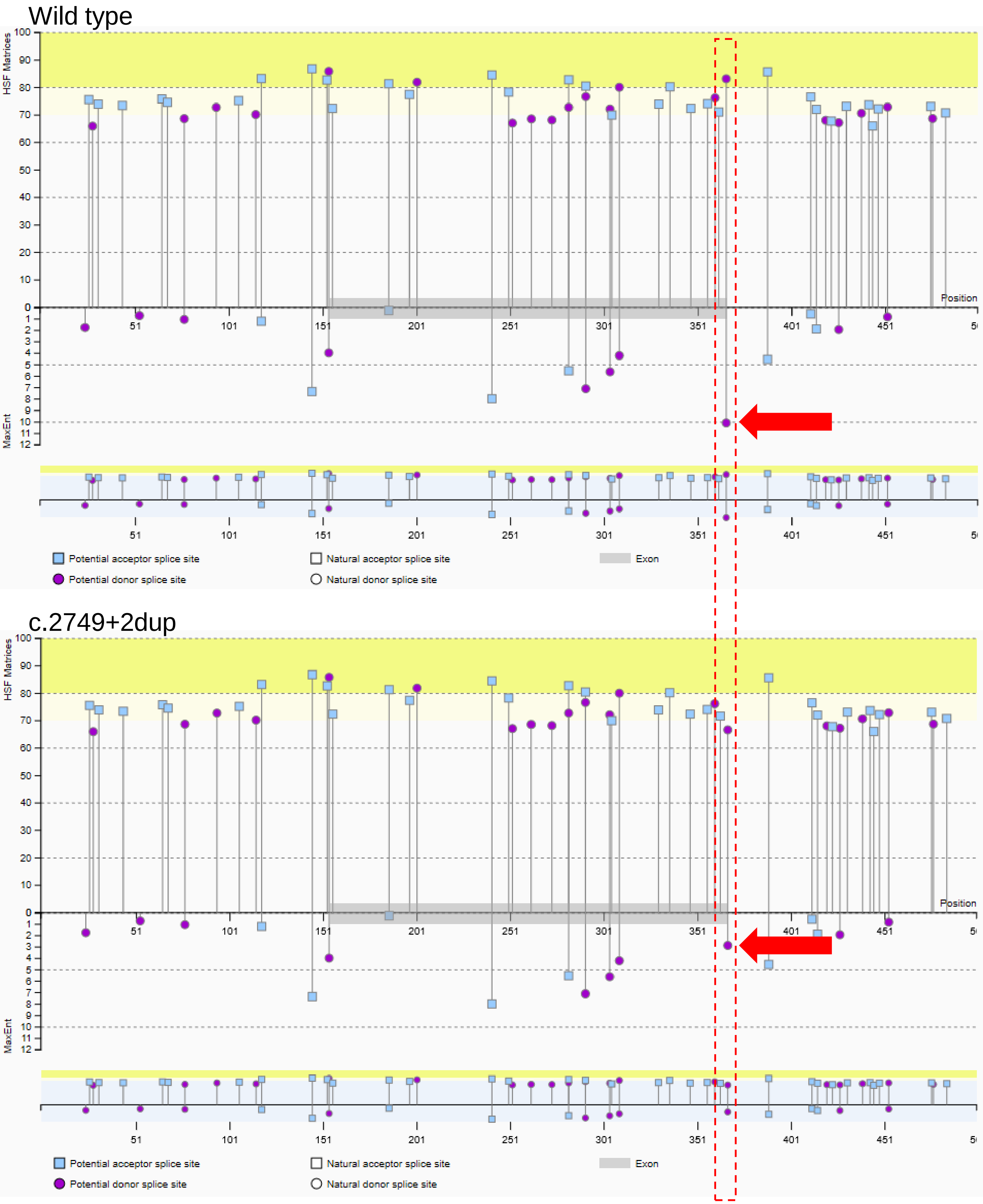

Exon 19 corresponds to the position 157-368. MaxEnt score (red arrows) is decreased by a 1-bp duplication, indicating loss of the original donor site (a dotted rectangle).

Supplemental Figure S2. In-silico analysis using ASSP

Wild type

Position (bp)	Putative splice site	Sequence	Score*	Intron GC*	Activations** Alt./Cryptic	Activations** Constitutive	Confidence**
96	Alt. isoform/cryptic donor	TGAAAAATATgtaaacattg	5.476	0.486	0.869	0.087	0.900
117	Alt. isoform/cryptic donor	CCCAACTGAGgtcccccaa	6.184	0.486	0.889	0.079	0.911
156	Alt. isoform/cryptic donor	CTTGCCTTAGgtgtgcagaa	8.648	0.500	0.833	0.121	0.855
157	Constitutive acceptor	cttgccttagGTGTGCAGAA	7.640	0.429	0.189	0.800	0.764
165	Alt. isoform/cryptic acceptor	aggtgtgcagAAGGCTATTT	2.656	0.486	0.605	0.379	0.374
253	Alt. isoform/cryptic acceptor	tccctggcagCTGTGACAGC	6.154	0.529	0.655	0.333	0.493
293	Alt. isoform/cryptic donor	TGTAAACCAGgtacaacagg	10.003	0.486	0.745	0.193	0.741
306	Alt. isoform/cryptic donor	CAACAGGCCGgtactgtgag	5.195	0.471	0.954	0.033	0.965
311	Alt. isoform/cryptic donor	GGCCGGTACTgtgagctctg	8.441	0.471	0.939	0.042	0.955
368	Constitutive donor	AACTGTCAGCgtaagtcctg	9.846	0.400	0.322	0.592	0.456
400	Alt. isoform/cryptic acceptor	cccctgacagAATTGATGTA	3.807	0.457	0.547	0.430	0.213
1251	Constitutive donor	AATTATTCAGgtgggtccca	13.214	0.414	0.147	0.803	0.817
1252	Constitutive acceptor	aattattcagGTGGGTCCCA	7.221	0.371	0.440	0.540	0.184

c.2749+2dup

Position (bp)	Putative splice site	Sequence	Score*	Intron GC*	Activations** Alt./Cryptic	Activations** Constitutive	Confidence**
96	Alt. isoform/cryptic donor	TGAAAAATATgtaaacattg	5.476	0.486	0.869	0.087	0.900
117	Alt. isoform/cryptic donor	CCCAACTGAGgtcccccaa	6.184	0.486	0.889	0.079	0.911
156	Alt. isoform/cryptic donor	CTTGCCTTAGgtgtgcagaa	8.648	0.500	0.833	0.121	0.855
157	Constitutive acceptor	cttgccttagGTGTGCAGAA	7.640	0.429	0.189	0.800	0.764
165	Alt. isoform/cryptic acceptor	aggtgtgcagAAGGCTATTT	2.656	0.486	0.605	0.379	0.374
253	Alt. isoform/cryptic acceptor	tccctggcagCTGTGACAGC	6.154	0.529	0.655	0.333	0.493
293	Alt. isoform/cryptic donor	TGTAAACCAGgtacaacagg	10.003	0.486	0.745	0.193	0.741
306	Alt. isoform/cryptic donor	CAACAGGCCGgtactgtgag	5.195	0.457	0.953	0.034	0.965
311	Alt. isoform/cryptic donor	GGCCGGTACTgtgagctctg	8.441	0.457	0.935	0.045	0.951
401	Alt. isoform/cryptic acceptor	cccctgacagAATTGATGTA	3.807	0.443	0.547	0.430	0.213
1252	Constitutive donor	AATTATTCAGgtgggtccca	13.214	0.414	0.147	0.803	0.817
1253	Constitutive acceptor	aattattcagGTGGGTCCCA	7.221	0.371	0.440	0.540	0.184

Exon 19 corresponds to the position 157-368. Due to a 1-bp duplication, constitutive donor site at 368 disappears (a dotted red rectangle). Alternatively, position 1252 may be used as a new donor site. This may extend exon 19 from 212-bp to 1096-bp. Blue dotted lines indicate omission.

Supplemental Figure S3. In-silico analysis using Fruit Fly Splice Predictor

Wild type

Donor site predictions

Start	End	Score	Exon Intron
150	164	0.41	gccttaggtgtgcag
287	301	0.94	aaaccaggtacaaca
362	376	0.99	tgtcagcgtaagtcc
539	553	0.82	taatagagtaaatac
1245	1259	0.96	tattcaggtgggtcc

c.2749+2dup

Donor site predictions

Start	End	Score	Exon Intron
150	164	0.41	gccttaggtgtgcag
287	301	0.94	aaaccaggtacaaca
540	554	0.82	taatagagtaaatac
1246	1260	0.96	tattcaggtgggtcc

Exon 19 corresponds to the position 157-368. Due to c.2749+2dup, splicing donor site at 362_376 indicated by red dotted rectangle disappears. Alternatively, positions 540_554 or 1246_1260 may be used as new donor sites in c.2749+2dup and exon 19 will be extended from 212-bp to 390-bp or 1096-bp, respectively. Both will cause frameshift.

Supplemental Figure S4. In-silico analysis using NetGene2

Wild type

```
Donor splice sites, direct strand
----------------------------------
             pos 5'->3'  phase strand  confidence  5'      exon intron    3'
                 294       1      +        0.41       TGTAAACCAG^GTACAACAGG
                 369       1      +        0.67       AACTGTCAGC^GTAAGTCCTG
                1252       1      +        0.34       AATTATTCAG^GTGGGTCCCA
```

c.2749+2dup

```
Donor splice sites, direct strand
----------------------------------
             pos 5'->3'  phase strand  confidence  5'      exon intron    3'
                 294       1      +        0.71       TGTAAACCAG^GTACAACAGG
                1253       1      +        0.34       AATTATTCAG^GTGGGTCCCA
```

Exon 19 corresponds to the position 157-368. Due to c.2749+2dup, splicing donor site at 369 indicated by red dotted rectangle disappears. Alternatively, positions 1253 may be used as a new donor site and exon 19 will be extended from 212-bp to 1096-bp, which will cause a frameshift.
